# University autonomy is a predictor of English medium instruction in European higher education

**DOI:** 10.1007/s10734-024-01333-8

**Published:** 2024-11-06

**Authors:** Peter Wingrove, Beatrice Zuaro, Marion Nao, Dogan Yuksel, Levente Littvay, Anna Kristina Hultgren

**Affiliations:** 1 Faculty of Wellbeing, Education and Language Studies, School of Languages and Applied Linguistics, The Open University, Milton Keynes, UK; 2 HUN-REN Centre for Social Sciences, Hungarian Academy of Science Centre of Excellence, Budapest, Hungary; 3 Democracy Institute in Budapest, Central European University, Budapest, Hungary

**Keywords:** Steering at a distance, University autonomy, English medium instruction, Higher education, Multilevel regression

## Abstract

Despite extensive research into English as a Medium of Instruction (EMI) in higher education, few if any studies have explored the role of higher education autonomy in driving EMI. This paper tests the novel hypothesis that university autonomy—spearheaded across European higher education through neoliberally predicated ‘steering at a distance’ reforms—predicts EMI. The data are multilevel with higher education institutions (HEIs) nested inside education systems. The University Autonomy Scorecards (Pruvot & Estermann, 2017) operationalise university autonomy at the education-system level (*n* = 26) and measure four dimensions of autonomy: academic, financial, staffing, and organisational. We include ‘overall autonomy’ as the average. The European Tertiary Education Register provides HEI-level data (*n* = 1815), which we combine with a count of English-taught degree programmes (ETPs) to measure EMI, provided by Study Portals, the largest online portal of degree programmes. We conduct multilevel regression to analyse the relationships between autonomy and EMI. The results showed that overall autonomy predicts EMI in public universities (*p* = 0.002). Increasing overall autonomy by one point above the mean increases the likelihood of offering EMI by 9.5%. Academic, staffing, and organisational autonomy predict EMI in public universities, whereas financial autonomy does not. The first to quantify a relationship between university autonomy and EMI, this study offers new insights into how EMI comes about. By revealing a previously obscured interconnectedness between language shift and higher education governance, the study demonstrates the value added of an interdisciplinary approach and proposes a new line of inquiry for future research.

## Introduction

The use of English to teach academic subjects mostly in higher education contexts, commonly referred to as ‘English-medium instruction’ (EMI), has been described as an ‘unstoppable train’ (Macaro, 2015). Studies which have investigated the presence of EMI in European higher education have reported rapid growth since the turn of the millennium, estimating up to tenfold growth in English-taught degree programmes (ETPs) in Europe between 2001 and 2014 (Wächter & Maiworm, 2014) and claiming up to 50-fold growth in English-taught bachelor’s programmes between 2009 and 2017 (Sandström & Neghina, 2017).^1^

To date, explanations for the growth of EMI in European higher education have largely pointed to The Bologna Process and internationalisation (Dimova et al., 2015; Hultgren, 2024; Macaro, 2018; Wilkinson & Gabriëls, 2021). In this paper, we propose a novel explanation, namely that EMI is driven by a set of neoliberal reforms that have granted higher education institutions (HEIs) greater autonomy and accountability. Whilst governance reforms are an established area of interest in fields such as higher education studies, public administration, political science, organisational studies, etc. (Dougherty & Natow, 2020; Kelchen, 2019), little attention has been paid to the relationship between governance and language of instruction.

One reason the relationship between governance and EMI has not been addressed in the literature is that political science and public administration are rarely concerned with language. Meanwhile, applied linguistics rarely engages substantially with higher education governance (Macaro & Aizawa, 2022). To bridge this gap, we approach the research question of what drives EMI through an interdisciplinary lens that bridges applied linguistics and political science and related fields. A second reason this topic has not been addressed concerns the availability of data which measure EMI. Major databases, such as Eurostat and OECD, typically collect data on education trends concerning participation, graduation, staffing, and finance, but language of instruction is a blind spot.

The question of what drives English is important for ethical, legal, and political reasons. An ethical concern is that whilst converging on a common language has benefits for international communication, defaulting to English can disadvantage non-native speakers, resulting in negative experiences for lecturers and students (Block, 2022). Furthermore, the primacy of English can result in ‘domain loss’: the erosion of other languages in academic and professional domains. In Italy, domain loss became a legal concern when lecturers at the Polytechnic University of Milan brought a case against the university when it decided to teach all MA and PhD degree programmes exclusively in English (Murphy & Zuaro, 2021). The case reached the constitutional court, and the university ultimately lost the appeal as teaching exclusively in English was deemed unconstitutional, threatening the primacy of the Italian language. A more recent development in the Netherlands has seen MPs vote on an education bill to require two-thirds of content for standard bachelor’s degrees to be in Dutch.^2^ This pushback may have come about as the Netherlands has been leading the way in EMI since the 1980s (Wilkinson Gabriëls, 2021) with 29% of bachelor’s degrees and 75% of master’s degrees taught exclusively in English by 2022 (VSNU, 2023). In response to the bill, the Universities of the Netherlands Association spokesperson argued that ‘this national control … interferes with the autonomy of universities’^2^. In both Italy and the Netherlands, EMI in higher education developed unchecked until it ran into legal or political resistance.

In this paper, we hope to bring some clarity to the connection between EMI and higher education autonomy, which has roots in neoliberal reform. Since the 1980s and onwards, most European countries have sought to reform their higher education systems towards granting institutions greater autonomy while putting into place more accountability, referred to as ‘steering at a distance’ (Kickert, 1995). The rationale behind such ‘steering at a distance’ approaches to governance is both economic and ideological (Eurydice, 2000). Economically, HEIs have faced pressure due to massification and a growing number of students pursuing university studies (Trow, 2006), necessitating more cost-effective modes of financing. Ideologically, steering at a distance reforms are premised on a neoliberal philosophy, traceable to the Reagan/Thatcher era in the 1980s, where a widespread distrust of etatism emerged alongside an associated belief that public sector institutions were inefficient and needed reform (Krüger et al., 2018; Capano & Pritoni, 2020). Steering at a distance reforms thus aimed to make HEIs more dynamic, competitive, and cost-effective (Bleiklie, 2018; Ferlie et al., 2008) by granting them greater autonomy and incentivising them to be proactive (Capano & Pritoni, 2020; Krüger et al., 2018).

Although ‘autonomy’ is a notoriously elusive concept, it is generally understood as independence from the state (Capano & Pritoni, 2020; Krüger et al., 2018). However, although government control has loosened in some areas, it has tightened in others (Enders et al., 2013; Euridyce, 2000) because the granting of autonomy has often been combined with the introduction of accountability mechanisms, such as ‘performance indicators’ (Minassians, 2015), ‘governance by numbers’ (Shore & Wright, 2015; Supiot, 2017), and ‘big data governance’ (Beerkens, 2021). In these modes of governance, the government sets targets for HEIs and then assumes (or delegates) the role of monitoring and assessing compliance, as captured in the rise of the ‘audit culture’ (Shore & Wright, 2015) and the ‘evaluative state’ (Neave, 2012). There has been a shift, in other words, from ‘government to governance’ (Eurydice, 2000; Krüger et al., 2018; Capano & Pritoni, 2020), with state power devolving in three directions (Pierre & Peters, 2020): upwards to supranational actors such as the OECD and the European Union; downwards to provinces, local governments, and HEIs themselves; and outwards to international higher education organisations such as the European Association for Quality Assurance in Higher Education, the Academic Cooperation Association, and not least the European University Association, whose data on autonomy we draw on in this study. With all this monitoring in place, some have argued that ‘autonomy’ has decreased, and the role of the nation-state has *changed* rather than *lessened* (Westerheijden et al., 2010). In our terms, autonomy in this context can be understood as a form of regulated autonomy, as opposed to a model of libertarian autonomy, which would occur in the absence of any government involvement whatsoever.

Methodologically, this paper uses multilevel regression to model the relationship between institutional autonomy and EMI in a cross-sectional study. EMI is measured at the level of HEIs in terms of English-taught degree programmes (ETPs). Our data includes 1815 HEIs, nested within 26 European education systems, with data on ETPs collected from Study Portals, the largest online portal of ETPs. Autonomy is measured at the level of education systems (which in most cases constitutes a country) by reference to the University Autonomy Scorecards (Pruvot & Estermann, 2017).

These scorecards measure institutional autonomy in European higher education across four dimensions: academic, financial, staffing, and organisational autonomy. Additionally, scores consider negotiations between institutions and the state on the use of accountability mechanisms. Taking the interplay of autonomy and accountability mechanisms into consideration aligns the scorecards more closely with ‘regulated’ autonomy in the context of steering at a distance reforms.

We theorise that autonomy is linked to EMI via constituting a part of, or a proxy for, the competitive environment brought about by steering at a distance reforms. It is within the globally competitive environment that HEIs are incentivised to transition from being primarily nationally to internationally oriented. This international competition necessitates a common language, English, which is adopted due to its status as a global *lingua franca*. Take the example of performance-based funding targets on enrolment (Kelchen, 2019). The targets themselves are an accountability mechanism and the universities are granted sufficient autonomy to meet these goals as they see fit. Universities can then use their given autonomy to implement EMI and EMI-facilitating policies, such as establishing new degree programmes in English, recruiting international staff, and setting internationally competitive tuition fees, which would enable them to meet enrolment (and other) targets.

From this perspective, autonomy may be a component in a causal chain that leads to EMI, moderating the effect of globalisation pressures in an English as a *lingua franca* environment on the provision of EMI. Our measure of autonomy, whilst perhaps a proxy for an internationally competitive environment, may also be a part of it, which enables universities to offer EMI as part of an internationalisation strategy. Although this study only investigates association not causation, we can speculate on the nature of this relationship as potentially causal, with perhaps global economic forces driving EMI, but autonomy facilitating it. Moreover, it is worth pointing out that autonomy itself, whether defined as regulated or libertarian, enables institutions to act within their own interests, which may or may not align with those of the state, or even their own faculty, as exemplified in the case of the Polytechnic of Milan.

Therefore, in this study, we investigate the relationship between autonomy and the ownership structure of universities in terms of ETPs. We suggest that, as steering at a distance reforms regulate the public sector, the effect of autonomy within an education system can be expected to affect public universities directly and private universities indirectly, potentially through competition with public universities. Prior theory suggests that competition between public and private universities may drive EMI (Macaro, 2018). This operates within a global market, where HEIs compete for students and staff. Whilst this complex interplay is hard to capture, we anticipate more EMI-practising public universities in systems which grant them greater institutional autonomy.

We test the following hypotheses. In public universities, is English-medium instruction predicted by:

1. Overall autonomy?
2. Academic autonomy?
3. Financial autonomy?
4. Staffing autonomy?
5. Organisational autonomy?

Here, ‘overall autonomy’ is the arithmetic mean of the four dimensions. This paper is structured as follows: data and measurements, results, discussion, and conclusion.

## Data and measurements

### Overview

This study combines multiple data sources into a two-level hierarchical data structure. In total, 1815 HEIs (meso-level) are nested inside 26 education systems (macro-level). Our meso-level data combines data on ETPs from Study Portals, the largest online portal of ETPs; with the European Tertiary Education Register (ETER; Lepori et al., 2023), a comprehensive database of European HEIs. Our macro-level data is comprised of the university autonomy scorecards provided by the European University Association (Pruvot & Estermann, 2017) and the Education First English Proficiency Index (EF EPI) (Education First, 2018). We base our analysis on the 2017 edition of the autonomy scorecards with ETPs commencing in the academic year 2018, L1 ETER data from 2018, and the EPI report from 2018. Therefore, this is a cross-sectional empirical study on the effect of autonomy on commencing ETPs in the following year.

### Meso-level data

As previously noted, our meso-level data (L1) are comprised of institutional data from ETER and data on ETPs from Study Portals. By joining Study Portals data (HEIs which offer EMI) with ETER (a comprehensive database of European HEIs), we avoid selecting on the dependent variable. In other words, if we only look at HEIs which offer EMI, this would overestimate the prevalence and success of EMI programmes by only looking at successful cases. Moreover, both databases cover the European Higher Education Area, which enables us to run our analyses across the entire region, rather than combining data from national databases, which likely vary in collection techniques and definition of EMI.

ETER provides a comprehensive database of European HEIs alongside meso-level predictors (L1). Our meso-level predictors comprise three classification indicators relevant to running EMI programmes: institutional control, institution size, and education intensity. We summarise these details from the ETER handbook (Lepori, 2023) as follows. Institutional control is a binary categorical variable, 0 if the HEI is private and mostly funded by private sources, and 1 if the institution is under public control or mostly funded by the state. Institution size is a categorical variable based on the number of full-time equivalent (FTE) academic staff. In cases of missingness, head count (HC) of academic staff is used to estimate FTE academic staff. There are four levels to institution size: (1) below 100 FTEs; (2) below 500 FTEs; (3) below 1500 FTEs; and (4) more or equal to 1500 FTEs. Education intensity is the number of ISCED 5–7 students divided by academic staff and sorted into four categories: (1) below 10 students per academic staff member; (2) below 25; (3) below 50; and (4) 50 or above. One strength of using ETER in our analysis is that these variables are consistently defined and measured across the countries in our study.

We include institutional control as our hypotheses concern the effect of autonomy on *public* HEIs in terms of ETPs. Institution size is included as our dependent variable is a count of ETPs not a percentage and therefore we are required to control for size. Education intensity accounts for the fact that institutions differ in pedagogic focus.

We joined data from Study Portals on the number of English-taught bachelor’s and master’s programmes commencing in a given academic year to ETER. The datasets were joined manually using detailed information on HEI name and location present in both datasets. As our hypotheses concern the effect of autonomy on HEIs in terms of EMI, represented by both bachelor’s and master’s ETPs, we analyse a combined response variable of the sum of master’s and bachelor’s ETPs. The exploration of multiple dependents divided by level or discipline is beyond the scope of a single paper. As this measure comes from an online portal, strictly speaking, this measure is a count of *internationally advertised* ETPs, which differs from some prior studies which collected data from institutional surveys (e.g. Wächter & Maiworm, 2008). The trade-offs between both measures are discussed in the ‘Limitations’ section.

**Fig. 1:**
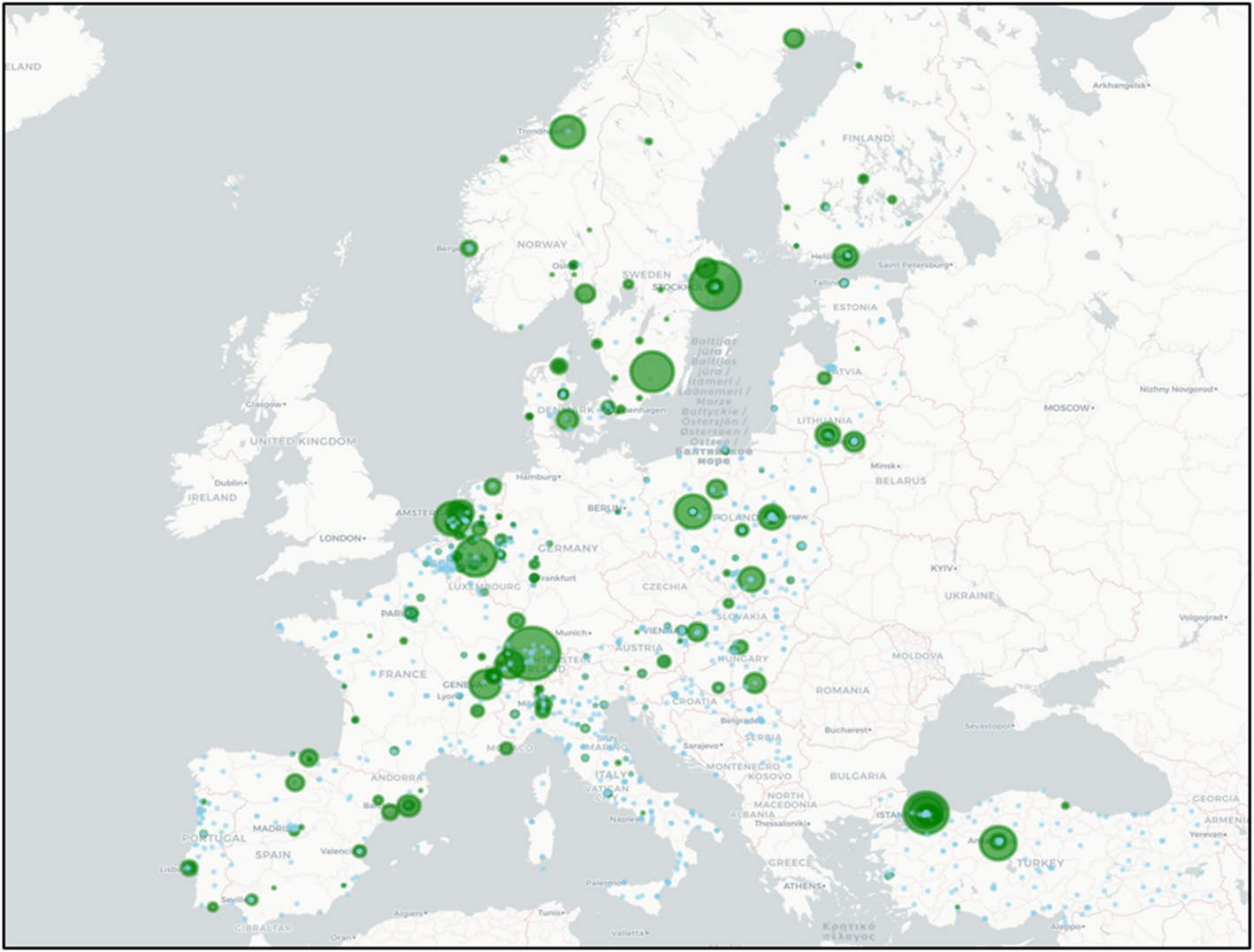
EMI in universities nested within European education systems in 2018

The HEIs represented in Study Portals data are a subset of ETER HEIs. ETER HEIs without programmes listed on Study Portals are allocated zero ETPs as default. Therefore, each institution starts at zero and we count each time an ETP is advertised. Figure 1 visualises these data, with green dots representing the count of ETPs. Note that regions without autonomy scorecards are not represented.

### Macro-level data

As noted in the overview, our macro-level (L2) data are comprised of 26 education systems from two sources: the university autonomy scorecards and the English Proficiency Index. All education systems represent a single country with two exceptions: Germany and Belgium. Due to the unique systems in these countries, three regional education systems (Brandenburg, Hessen, and North Rhine-Westphalia) are represented from Germany and two regional education systems (Flanders and Wallonia-Brussels Federation) are represented from Belgium. However, we maintain a two-level hierarchical structure.

The scorecards operationalise university autonomy and enable us to test our hypotheses. Autonomy is measured across four dimensions, each determined by indicators (Table 1). The scorecards measure each dimension on a scale of 0–100; however, ostensibly scores range from 32 to 100, with average dimension scores ranging from 59 to 69. Scores are calculated by starting from 100 and deducting points based upon restrictions to indicators, which are weighted by importance. ‘Overall autonomy’ is the arithmetic mean of all four dimensions.

**Table 1:** Dimensions of university autonomy (Pruvot & Estermann, 2017)

Academic	Financial
- Capacity to decide on overall student numbers	- Length and type of public funding
- Ability to select students	- Capacity to keep surplus
- Ability to introduce programmes	- Capacity to borrow money
- Ability to terminate programmes	- Ability to own buildings
- Ability to choose language of instruction	- Ability to charge tuition fees for national/EU students
- Capacity to select QA mechanisms and providers	- Ability to charge tuition fees for non-EU students
- Ability to design content of degree programmes	
Staffing	Organisational
- Ability to decide on recruitment procedures	- Selection procedure for the executive head
- Ability to decide on salaries	- Selection criteria for the executive head
- Ability to decide on dismissals	- Dismissal of the executive head
- Ability to decide on promotions	- Term of office of the executive head
- Note: indicators pertain to senior academic and administrative staff	- Inclusion and selection of external members in governing bodies
	- Capacity to decide on academic structures
	- Capacity to create legal entities

The 2017 edition of the autonomy scorecards acts as a predictor for ETPs commencing in 2018. These programmes can most likely be traced to prior years in terms of administration, which puts their inception in 2017 or perhaps earlier. This likely varies between institutions and is unknowable from the perspective of a large-scale generalisable study. Likewise, the time taken for macro-level policies to effect meso-level outcomes is not measured by our study. Instead, this study provides a generalisable look at the state of autonomy as it stands in 2017 and whether this predicts commencing ETPs in the following year. Pin-pointing when events occurred and how they are related is beyond the remit of our predictive modelling.

The English Proficiency Index captures the average level of English ability for each level 2 group through test scores. In the 2018 edition, scores are measured between 0 and 100 based upon test data from over 1,300,000 test-takers worldwide. This measure acts as a control with two competing hypotheses: greater English language competence predicts more ETPs as the population is more amenable to EMI. Alternatively, lower English language ability predicts more ETPs as English language skills are in demand. In either case, we have theoretical reasons that include EPI as a relevant predictor.

### Data imputation

We conducted data imputation to address missingness in the data. We used predictive mean matching offered by the mice package (v3.16.0; van Buuren & Groothuis-Oudshoorn, 2011) in R to impute data for our L1 variables in the ETER database. Prior studies have suggested the value of imputation with this dataset (Bruni et al., 2021). For our L2 variables, we imputed data taking the average between subsequent and prior years. For instance, we imputed EPI scores for Estonia and Latvia in 2018, as they were not included in that edition.

### Analysis

We test our hypotheses concerning the 2017 edition of the autonomy scorecards using data on ETPs commencing the following academic year. The two most recent autonomy scorecards were published in 2017 and 2023; however, the ETER project presents data from 2010 to 2020, requiring us to base our analysis on the 2017 scorecard.

In total, 1815 HEIs are nested within 26 education systems. As our data and hypotheses are multilevel, we use multilevel regression to test the relationship between institutional autonomy and EMI. Our dependent variable is zero-inflated and over-dispersed. Therefore, we fit zero-inflated negative binomial models.^3^ Models that ignore zero-inflation or treat it as overdispersion can bias parameter estimates (Harrison, 2014). Zero-inflated negative binomial (ZINB) models effectively run two models simultaneously: a logistic regression (the zero-inflation model), which estimates the probability of observing excess zeros; and a negative binomial regression (the conditional model), which models the count of ETPs.

The zero-inflation model predicts the probability of observing excess zeros. That is, zeros not due to the count distribution in our conditional model (‘sampling zeros’), but zeros which occur for structural reasons (‘excess zeros’), which would indicate HEIs not practicing EMI at all. For instance, a daily count of alcohol use would contain sampling zeros on days when individuals do not drink alcohol. However, teetotal individuals would have structural zeros for all observations. Similarly, some institutions may not practice EMI at all, whereas others may practice EMI but still have sampling zeros. By fitting a ZINB model, we avoid biasing our estimates and we are able to (1) predict EMI versus non-EMI institutions (zero-inflation) and (2) predict the count of ETPs in EMI-practicing institutions (conditional).

Concerning interpretation, a negative estimate in the zero-inflation model indicates a reduction in the likelihood of observing excess zeros, i.e. an *increased* likelihood of observing EMI. The conditional model is the opposite: a negative estimate predicts a *reduction* in the number of ETPs in EMI-practising HEIs.

Our model building process started with intercept-only models, and we added theoretically relevant predictors which increased model fit. The result of our model building process is formulated as follows:

**Zero-inflation model:
**

- Level 1: $$logit(ETPs)ij= \beta 0j+ \beta 1j\left(Public\right)ij+rij$$
- Level 2: $$\beta 0j= \gamma 00+ \gamma 01\left(Autonomy\right)j+ \gamma 02(EPI)j$$
- $$\beta 1j=\gamma 10+ \gamma 11(Autonomy)j$$

**Conditional model:
**

- Level 1: $$count\left(ETPs\right)ij= \beta 0j+ \beta 1j\left(Public\right)ij+\beta 2j\left(Controls\right)ij +eij$$
- Level 2: $$\beta 0j= \gamma 00+ \gamma 01(Autonomy)j+ \gamma 02(EPI)j+u0j$$
- $$\beta 1j=\gamma 10+ \gamma 11(Autonomy)j+u1j$$
  $$\beta 2j= \gamma 20$$

**Correlation:
**

$$\rho ij(eij, rij)$$

In the zero-inflation model, the dependent variable is the log-odds of observing excess zeros for the *i*th HEI within the *j*th education system. β0*j* represents the intercept. ‘Public’ indicates whether the HEI is public (1) or private (0). At level 2, ‘autonomy’ represents the autonomy scorecards, either as overall autonomy or as specific dimensions. The English Proficiency Index is denoted by EPI.

In the conditional model, the dependent variable is the predictive log of the expected count of ETPs. This contains ‘controls’ relevant to the count distribution: institution size and education intensity. As ‘public’ forms the lower-level variable in a cross-level interaction, it is included as a random slope (Heisig & Schaeffer, 2019). We centred our continuous variables. The correlation parameter, ρ*ij*, accounts for dependence between the conditional and zero-inflation models.

## Results

### Overall autonomy

Starting with hypothesis 1: is overall autonomy a predictor of English-medium instruction in public universities? In Table 2, the main effect of overall autonomy shows the effect of autonomy on private HEIs (i.e. assuming ‘public’ is 0) and the interaction term shows how the effect of autonomy differs between public and private HEIs. In the logistic model, where a negative coefficient predicts fewer excess zeros (i.e. more EMI-practising HEIs), the effect of being a public university is negative and non-significant (coefficient =  − 1.59, standard error = 0.902, *p* = 0.078), suggesting that there is no difference between public HEIs and private HEIs, everything else held constant. The fact that this result is close to significance suggests that public HEIs *may* be more likely to offer EMI, but we cannot reject the null hypothesis that there is no effect.

**Table 2:** Overall autonomy as a predictor of ETPs

	**Overall autonomy**			
***Zero-inflation fixed effects (logistic)***	***Coef***	***SE***	***OR***	***p***
Level 1				
Intercept	− 0.114	0.677	0.892	0.866
Public	− 1.590	0.902	0.204	0.078
Level 2				
English proficiency	− 0.125	0.068	0.882	0.064
Overall autonomy	0.050	0.043	1.051	0.247
Cross-level interaction				
Public × overall autonomy	− 0.150	0.049	0.861	0.002**
***Conditional fixed effects (negative binomial)***	***Coef***	***SE***	***IRR***	***p***
Level 1				
Intercept	− 4.497	0.555	0.011	< 0.001***
Size	1.535	0.108	4.642	< 0.001***
Education intensity	0.320	0.121	1.377	0.008**
Public	− 0.224	0.453	0.799	0.621
Level 2				
English proficiency	− 0.021	0.050	0.979	0.678
Overall autonomy	0.017	0.035	1.017	0.628
Cross-level interaction				
Public × overall autonomy	0.010	0.037	1.010	0.798
***Conditional random effects (negative binomial)***	***Var***	***SD***	***Corr***	
Intercept	0.714	0.845		
Public *slope*	1.094	1.046	− 0.59	
***Model fit***	***R***^2^(Cond.)	***R***^2^(Marg.)	***AIC***	
	0.49	0.35	2530	

Observations: 1815; groups: 26

Significance: ***p > 0.001; **p > 0.01; *p > 0.05

We find that the effect of overall autonomy (on private HEIs) is positive and non-significant (coefficient = 0.050, standard error = 0.043, *p* = 0.247), suggesting there is no effect. However, the interaction shows that the effect of autonomy is dependent on whether the HEI is public or private. Here we find a negative and significant result (coefficient =  − 0.15, standard error = 0.049, *p* = 0.002). Therefore, there is no difference between public or private HEIs, except when we increase overall autonomy, in which case we find that public HEIs are more likely to offer EMI compared to private HEIs. This confirms our hypothesis that overall autonomy is a significant predictor of EMI in public universities.

**Fig. 2:**
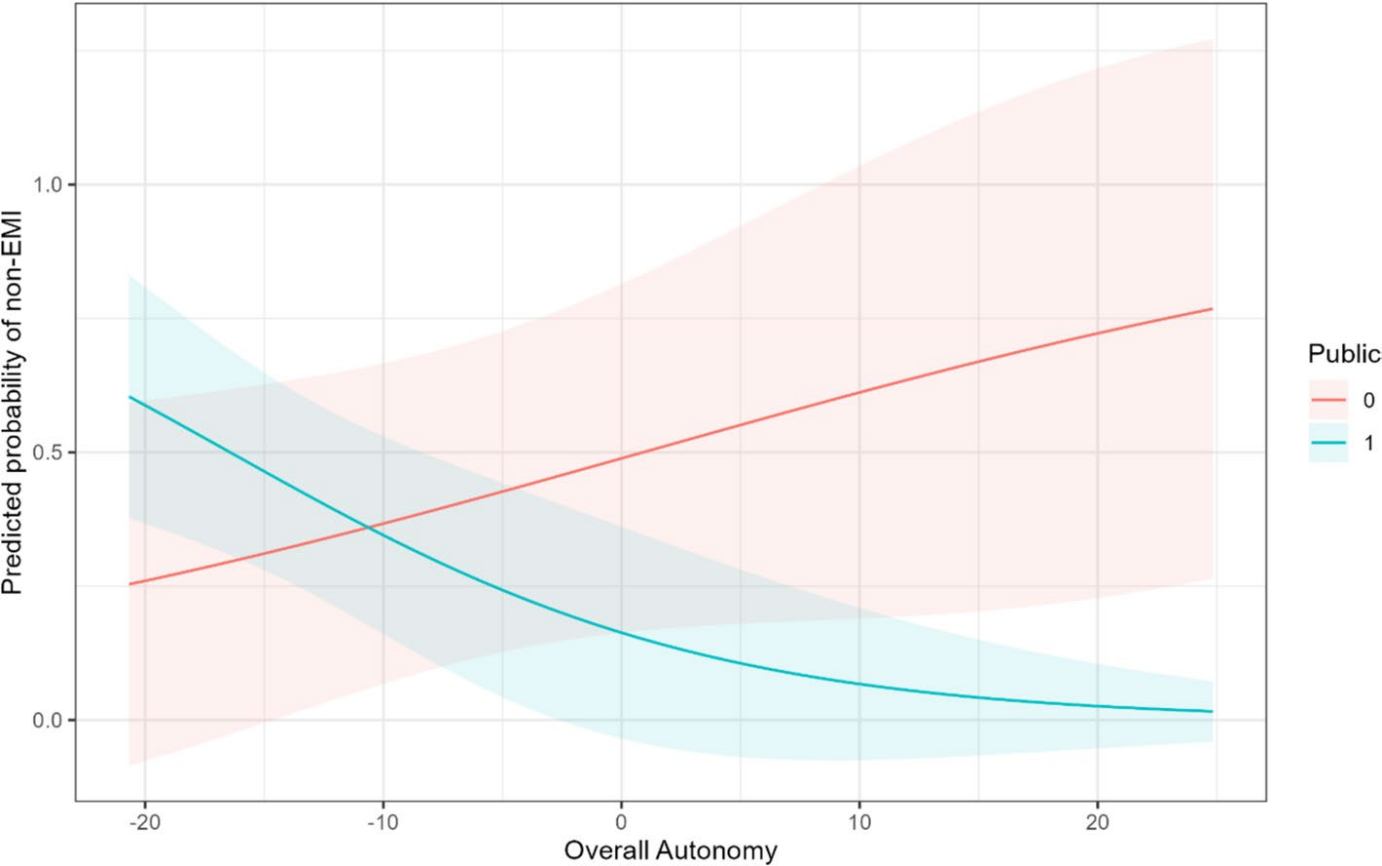
Predicted probabilities of non-EMI HEIs (excess zeros) by overall autonomy

Concerning effect size, the odds ratio (OR) for the effect of overall autonomy within public universities^4^ suggests that increasing overall autonomy by one point above the mean, whilst controlling for all other variables, increases the likelihood of public universities offering EMI by 9.5%. If we increase overall autonomy by ten points, the likelihood of offering EMI increases by 63%. We visualise the predicted probabilities in Fig. 2, which shows that as overall autonomy increases, the likelihood of observing excess zeros decreases.

In the negative binomial model, a positive coefficient predicts a greater number of ETPs in EMI-practising HEIs. Here we find non-significant results concerning whether the institution is public or private (coefficient =  − 0.224, standard error = 0.453, *p* = 0.621), the effect of overall autonomy (on private HEIs) (coefficient = 0.017, standard error = 0.035, *p* = 0.628), and how the effect of autonomy differs between public and private in the interaction (coefficient = 0.01, standard error = 0.037, *p* = 0.798). The directionality of the effect suggests that increasing overall autonomy may result in an increased number of ETPs in public EMI-practising institutions, although the evidence is not strong enough to reject the null hypothesis that there is no effect.

**Fig. 3:**
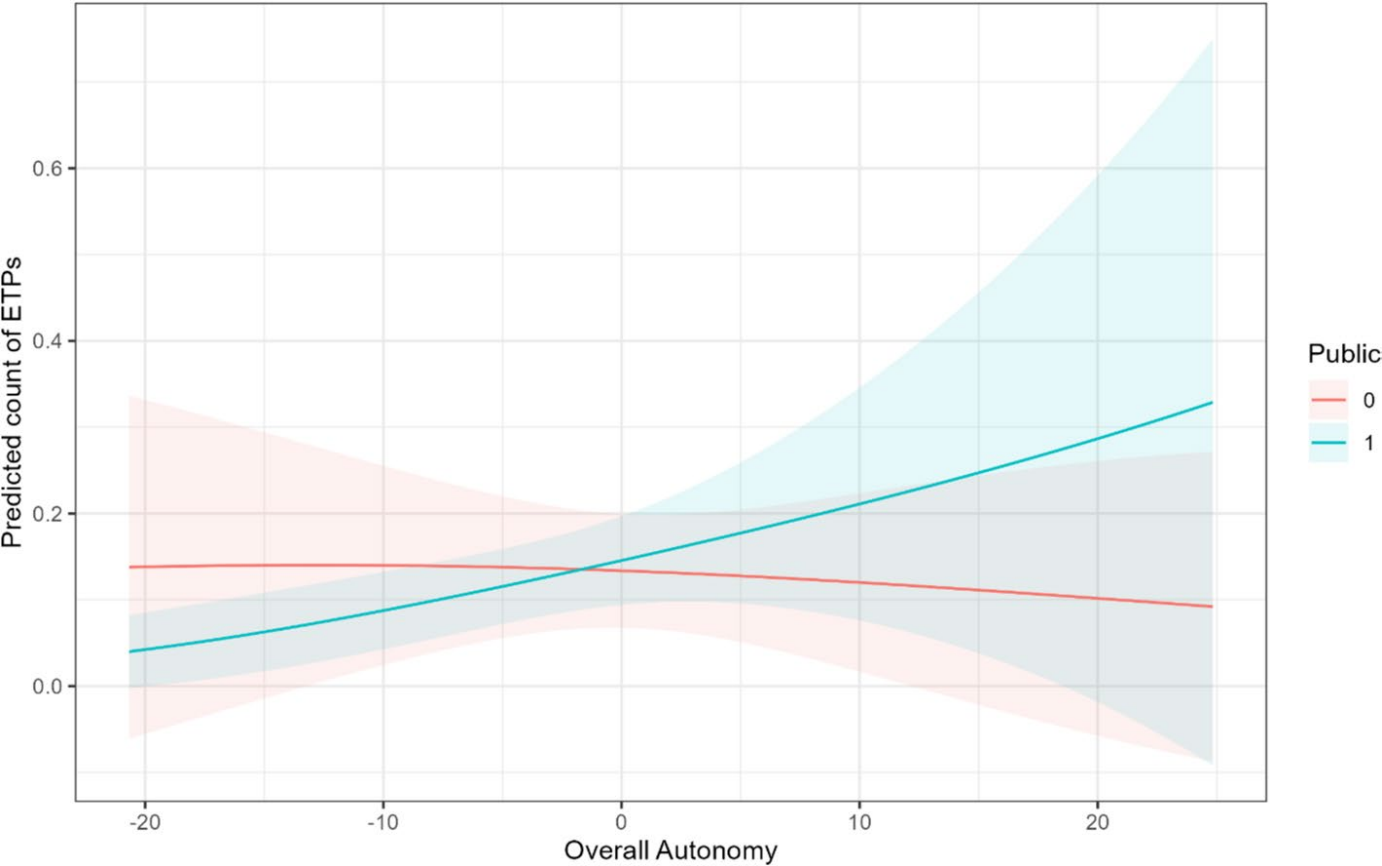
Predicted count of ETPs by overall autonomy

As a measure of effect size, the incident rate ratio (IRR) suggests that the number of ETPs will be 2.7% larger in public HEIs that have one more point of overall autonomy compared to public HEIs of an equal size, of equal pedagogic focus, and with an equal level of English ability within the country. An increase of ten points of overall autonomy above the mean suggests that the number of offered ETPs will be 30.1% larger. We visualised the predicted count of ETPs in Fig. 3, which shows (with great uncertainty) that as overall autonomy increases, the predicted count of ETPs increases in public HEIs.

### Dimensions of autonomy

**Fig. 4:**
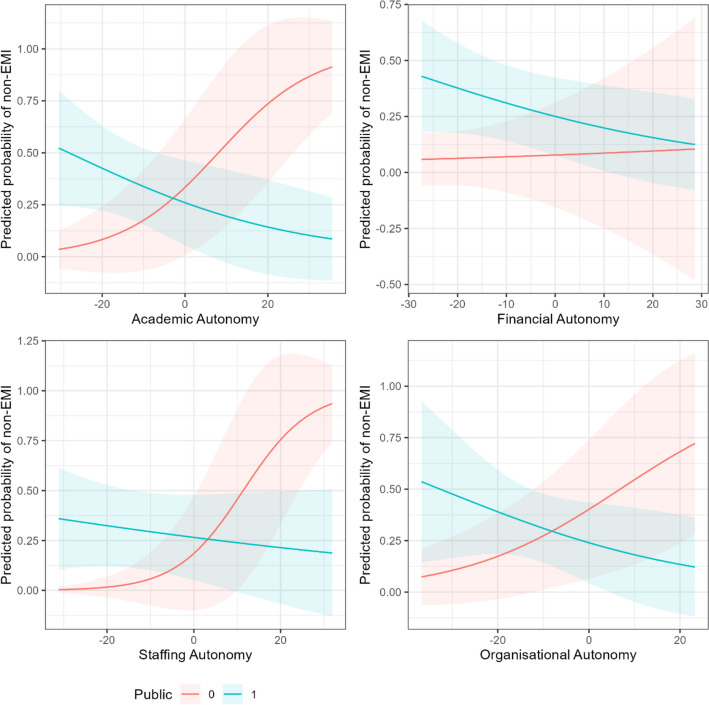
Predicted probabilities of non-EMI HEIs (excess zeros) by dimension of autonomy

**Fig. 5:**
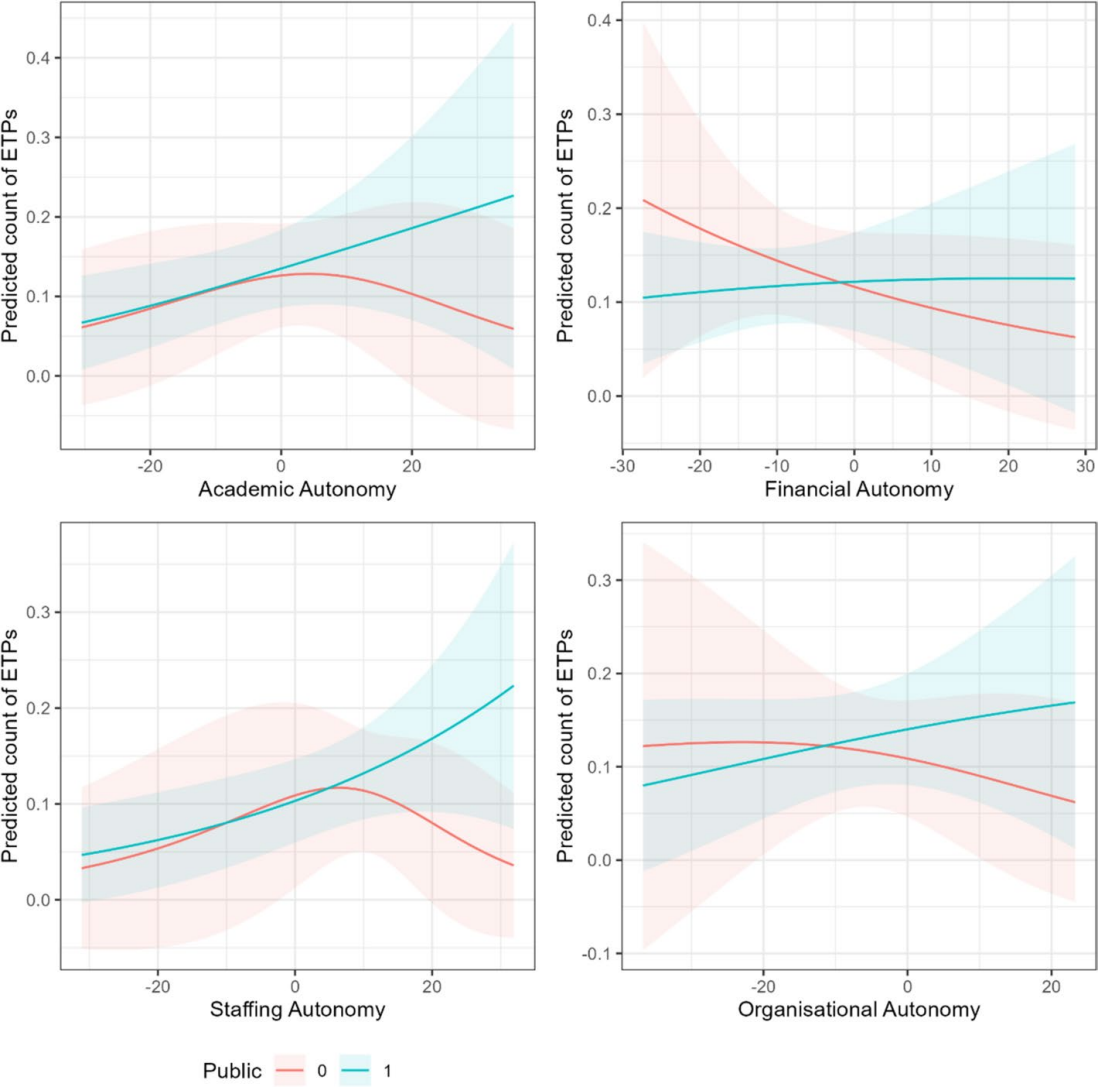
Predicted count of ETPs by dimension of autonomy

Now that we have established that overall autonomy is a predictor of EMI in public universities, does this hold concerning the individual dimensions of autonomy? In this section, we show that academic, staffing, and organisational autonomy predict EMI, but financial autonomy does not (zero-inflation models). We find insignificant results concerning whether EMI-offering institutions offer more EMI (conditional models). We visualise the predicted probabilities of excess zeros in Fig. 4 and the predicted count of ETPs in Fig. 5.

Academic autonomy concerns the pedagogic activities of the university. Table 3 shows that academic autonomy is a significant predictor of EMI in public HEIs compared to private HEIs in our zero-inflation model (coefficient =  − 0.123, standard error = 0.039, *p* = 0.002). We find an insignificant result concerning the effect of being a public HEI. Therefore, being a public or private HEI does not predict an increased probability of offering EMI; however, when we increase academic autonomy, offering EMI becomes more likely in public HEIs. Interestingly, we find a significant result concerning the main effect of academic autonomy (on private HEIs) suggesting that increasing academic autonomy increases the likelihood of private HEIs *not* offering EMI. This suggests that increasing academic autonomy has opposite effects on private versus public HEIs. In terms of effect size, the odds ratio suggests that increasing academic autonomy one point above the mean increases the likelihood of public HEIs offering EMI by 3.7%, which increases to 31.1% for a ten-point increase. The IRR for the interaction in the conditional model suggests that the number of ETPs will be 0.9% larger in public HEIs with a one-point increase of academic autonomy, and 1.5% larger for a ten-point increase.

**Table 3:** Academic autonomy as a predictor of ETPs

	**Academic autonomy**			
***Zero-inflation fixed effects (logistic)***	***Coef***	***SE***	***OR***	***p***
Level 1				
Intercept	− 0.804	0.777	0.447	0.301
Public	− 0.355	0.731	0.701	0.627
Level 2				
English proficiency	− 0.209	0.066	0.811	0.002**
Academic autonomy	0.086	0.035	1.089	0.015*
Cross-level interaction				
Public × academic autonomy	− 0.123	0.039	0.884	0.002**
***Conditional fixed effects (negative binomial)***	***Coef***	***SE***	***IRR***	***p***
Level 1				
Intercept	− 4.584	0.542	0.010	< 0.001***
Size	− 0.077	0.434	4.558	< 0.001***
Education intensity	1.517	0.109	1.372	0.009**
Public	0.316	0.120	0.926	0.860
Level 2				
English proficiency	− 0.017	0.050	0.983	0.732
Academic autonomy	0.036	0.024	1.037	0.135
Cross-level interaction				
Public × academic autonomy	− 0.027	0.026	0.973	0.285
***Conditional random effects (negative binomial)***	***Var***	***SD***	***Corr***	
Intercept	0.800	0.894		
Public *slope*	1.310	1.145	− 0.67	
***Model fit***	***R***^2^(Cond.)	***R***^2^(Marg.)	***AIC***	
	0.491	0.352	2534	

Observations: 1815; groups: 26

Significance: ***p > 0.001; **p > 0.01; *p > 0.05

Financial autonomy concerns the financial freedom of the university. Table 4 shows that financial autonomy is not a predictor of EMI in public HEIs compared to private HEIs (coefficient =  − 0.041, standard error = 0.064, *p* = 0.518). However, the directionality of the effect suggests that increasing financial autonomy may increase the likelihood of public HEIs offering EMI compared to private HEIs, although we cannot reject the null hypothesis that there is no effect. In terms of effect size, the odds ratio suggests that increasing financial autonomy by one point above the mean increases the likelihood of public HEIs offering EMI by 2.9%, which increases to 25.7% for a ten-point increase. The IRR for the interaction in our conditional model suggests − 0.4% fewer ETPs in public HEIs for a one-point increase in financial autonomy above the mean, and − 4.4% fewer for a ten-point increase. Importantly, all these results are insignificant.

**Table 4:** Financial autonomy as a predictor of ETPs

	**Financial autonomy**			
***Zero-inflation fixed effects (logistic)***	***Coef***	***SE***	***OR***	***p***
L﻿evel 1				
Intercept	− 2.615	1.681	0.073	0.120
Public	1.375	1.536	3.957	0.371
L﻿evel 2				
English proficiency	− 0.258	0.064	0.773	< 0.001***
Financial autonomy	0.011	0.061	1.011	0.851
Cross-level interaction				
Public × financial autonomy	− 0.041	0.064	0.960	0.518
***Conditional fixed effects (negative binomial)***	***Coef***	***SE***	***IRR***	***p***
L﻿evel 1				
Intercept	− 5.157	0.491	0.006	< 0.001***
Size	1.558	0.109	4.747	< 0.001***
Education intensity	0.315	0.120	1.370	0.009**
Public	0.304	0.381	1.355	0.425
L﻿evel 2				
English proficiency	0.010	0.042	1.011	0.801
Financial autonomy	− 0.021	0.022	0.980	0.349
Cross-level interaction				
Public × financial autonomy	0.016	0.024	1.016	0.499
***Conditional random effects (negative binomial)***	***Var***	***SD***	***Corr***	
Intercept	0.942	0.971		
Public *slope*	0.870	0.933	− 0.59	
***Model fit***	***R***^2^(Cond.)	***R***^2^(Marg.)	***AIC***	
	0.480	0.347	2537	

Observations: 1815; groups: 26

Significance: ***p > 0.001; **p > 0.01; *p > 0.05

Staffing autonomy concerns the recruitment, promotion, dismissal, and salaries of senior administrative and senior academic staff. Table 5 shows that staffing autonomy is a significant predictor of EMI in public HEIs compared to private HEIs (coefficient =  − 0.145, standard error = 0.052, *p* = 0.005). We find a significant result concerning the main effect of staffing autonomy (on private HEIs) suggesting that increasing staffing autonomy decreases the likelihood of private HEIs offering EMI. In terms of effect size, the odds ratio suggests that increasing staffing autonomy by one point above the mean increases the likelihood of public HEIs offering EMI by 1.4%, which increases to 23.4% for a ten-point increase. The IRR for the interaction in our conditional model predicts 2.1% more ETPs in public HEIs for a one-point increase in staffing autonomy, and 23.4% more ETPs for a ten-point increase.

**Table 5:** Staffing autonomy as a predictor of ETPs

	Staffing autonomy			
***Zero-inflation fixed effects (logistic)***	***Coef***	***SE***	***OR***	***p***
L﻿evel 1				
Intercept	− 1.630	1.001	0.196	0.103
Public	0.467	0.814	1.595	0.567
L﻿evel 2				
English proficiency	− 0.267	0.083	0.765	0.001**
Staffing autonomy	0.131	0.052	1.139	0.013*
Cross-level interaction				
Public × staffing autonomy	− 0.145	0.052	0.865	0.005**
***Conditional fixed effects (negative binomial)***	***Coef***	***SE***	***IRR***	***p***
L﻿evel 1				
Intercept	− 4.675	0.534	0.009	< 0.001***
Size	1.531	0.110	4.622	< 0.001***
Education intensity	0.346	0.122	1.414	0.004
Public	− 0.072	0.403	0.930	0.858
L﻿evel 2				
English proficiency	− 0.032	0.052	0.968	0.538
Staffing autonomy	0.045	0.030	1.046	0.134
Cross-level interaction				
Public × staffing autonomy	− 0.024	0.030	0.977	0.422
***Conditional random effects (negative binomial)***	***Var***	***SD***	***Corr***	
Intercept	1.056	1.028		
Public *slope*	0.594	0.771	− 0.61	
***Model fit***	***R***^2^(Cond.)	***R***^2^(Marg.)	***AIC***	
	0.495	0.363	2533	

Observations: 1815; groups: 26

Significance: ***p > 0.001; **p > 0.01; *p > 0.05

Organisational autonomy concerns the leadership structure of the university. Table 6 shows that organisational autonomy is a significant predictor of EMI in public HEIs compared to private HEIs (coefficient =  − 0.093, standard error = 0.034, *p* = 0.007). The effect of organisational autonomy (on private HEIs) is also significant, suggesting that increasing organisational autonomy predicts fewer private HEIs offering EMI. In terms of effect size, the odds ratio for the interaction suggests that increasing organisational autonomy by one point above the mean predicts a 3.5% increase in the likelihood of public HEIs offering EMI, which increases to 48.2% for a ten-point increase. The interaction in the conditional model is insignificant, with the IRR suggesting a one-point increase of organisational autonomy above the mean predicting 0.2% more ETPs, which increases to 8.4% for a ten-point increase.

**Table 6:** Organisational autonomy as a predictor of ETPs

	Organisational autonomy			
***Zero-inflation fixed effects (logistic)***	***Coef***	***SE***	***OR***	***p***
L﻿evel 1				
Intercept	− 0.526	0.727	0.591	0.470
Public	− 0.756	0.820	0.470	0.357
L﻿evel 2				
English proficiency	− 0.233	0.068	0.792	0.001***
Organisational autonomy	0.058	0.028	1.060	0.035*
Cross-level interaction				
Public × organisational autonomy	− 0.093	0.034	0.911	0.007**
***Conditional fixed effects (negative binomial)***	***Coef***	***SE***	***IRR***	***p***
L﻿evel 1				
Intercept	− 4.608	0.564	0.010	< 0.001***
Size	1.521	0.110	4.576	< 0.001***
Education intensity	0.304	0.120	1.356	0.011*
Public	− 0.062	0.390	0.940	0.874
L﻿evel 2				
English proficiency	− 0.002	0.048	0.998	0.965
Organisational autonomy	0.009	0.027	1.009	0.751
Cross-level interaction				
Public × organisational autonomy	− 0.007	0.028	0.993	0.808
***Conditional random effects (negative binomial)***	***Var***	***SD***	***Corr***	
Intercept	1.262	1.123		
Public *slope*	0.404	0.635	− 0.65	
***Model fit***	***R***^2^(Cond.)	***R***^2^(Marg.)	***AIC***	
	0.464	0.312	2536	

Observations: 1815; groups: 26

Significance: ***p > 0.001; **p > 0.01; *p > 0.05

We can further our exploration by visualising the predicted probabilities of excess zeros by differing levels of autonomy between public and private HEIs (Fig. 4) and likewise the predicted count of ETPs (Fig. 5). Figure 4 shows how increases in academic, staffing, and organisational autonomy decrease the likelihood of observing excess zeros in public HEIs (i.e. observing EMI) and increase the likelihood of observing excess zeros in private HEIs (i.e. observing non-EMI). This difference is most stark concerning academic autonomy and staffing autonomy.

In Fig. 5, the weakness of our modelling in comparison to our zero-inflated models is apparent here, where we can see broad trends, which hint at an effect of increasing autonomy associated with a greater count of ETPs in public HEIs, but not strongly enough to reject the null hypothesis in each case that there is no effect.

## Discussion

Our results open up discussion questions: why is overall autonomy a predictor with the largest effect size? Why are academic, staffing, and organisational autonomy predictors of EMI, but financial autonomy is not? Why are our models better at predicting EMI versus non-EMI and worse at predicting the count of ETPs in EMI-practising HEIs? Key to this discussion is the role of institutional autonomy as a central component of ‘steering at a distance’ reforms in European higher education and the implications the current findings have for theorisations on the rise of English as the language of instruction in European higher education.

Overall autonomy was found to be a predictor of EMI (*p* = 0.002). Our model predicts that an increase of one point of overall autonomy above the mean increases the likelihood of public universities offering EMI by 9.5%, and ten points increases the likelihood by 63%. These predicted values are higher than those for the individual dimensions, suggesting that HEIs require freedom in multiple dimensions in order to offer EMI. Importantly, this finding provides statistical evidence for theories in linguistics that link the rise of English with neoliberalism. This is in light of a recent ‘political economy turn’ in applied linguistics (Block, 2017), focussing on English language teaching and learning within the context of neoliberalism (e.g. Block et al., 2013; Bori, 2018), where the English language itself is a form of capital (Petrovic & Yazan, 2021). This can be framed within a broader context whereby English has had a free ride on the back of global capitalism, going back as far as the 1600 s (O’Regan, 2021). The recent turn towards neoliberalism is the modern extension of this history and steering at a distance reforms within higher education are but one component of this complex behemoth.

Higher education expansion, which includes developing programmes, hiring staff, recruiting students, and generating revenue, involves endogenous processes: as it expands, it affects factors which affect expansion. The provision of EMI is situated within this ecosystem and this research does not make claims as to which event, the granting of autonomy and the provision of EMI, precedes another. While this study explored the extent to which granting HEIs autonomy increases their likelihood of offering ETPs, neither of these processes occurs in isolation but in tandem with myriads of other social processes.

There is, however, evidence from a case study in the Netherlands that illustrated how granting universities autonomy can encourage the provision of EMI. In a case university in the Netherlands, EMI was introduced only a couple of years after the country had gone through a steering at a distance reform in 1985. There is interview data that the enhanced autonomy and added accountability mechanisms that were put into place with the reform did incentivise the university management to introduce EMI at master’s level (Hultgren & Wilkinson, 2022). Elsewhere in Europe, however, where the rise of EMI is more recent, it is arguably more difficult to pinpoint which event precedes another and it is likely that ‘steering at a distance’ reforms and ‘EMI’ flow back and forth across countries in tandem with other globalisation processes.

In general, however, the introduction of a neoliberal type of higher education governance opens up the sector for increased competition, marketisation, and commodification. We suggest that part of this commodification involves the value placed upon EMI, enhancing the perceived value of a university degree, as students signal knowledge of their discipline, English ability, and a connection to international communities of scholars. Whilst this may be partly driven by students, the universities also benefit by attracting a wider talent pool of staff and students, increasing revenue, and climbing university rankings.

However, beyond overall autonomy, does the dimension matter? Our results suggest that academic, staffing, and organisational autonomy are most important. Academic autonomy concerns the pedagogic activities of the university. Academic autonomy was found to be a predictor of EMI (*p* = 0.002), with our model predicting that increasing academic autonomy by one point above the mean increases the likelihood of public HEIs offering EMI by 3.7%. A ten-point increase suggests an increased likelihood of 31.1%. To see why academic autonomy is important, we can turn to the specific criteria in question. Academic autonomy includes several key criteria for offering EMI, including ‘ability to choose the language of instruction’, ‘the ability to introduce and terminate degree programmes’, ‘the capacity to decide on overall student numbers’, and ‘the ability to select students’. Whilst the ability to choose the language of instruction and the ability to introduce/terminate programmes are self-explanatory, we can discuss the relevance of autonomy over students and admissions.

A key area of academic autonomy is related to selecting students and deciding upon overall student numbers. The relevance of these areas coheres with our re-theorising of the growth of EMI as linked to the internationally competitive environment brought about by steering at a distance policies. Universities use English programmes as means to attract a wider talent pool of international students. This coheres with Macaro’s (2018) suggestion that competition for students between public and private universities may be driving EMI growth. In this sense, public universities can only compete for students internationally if they are given the autonomy to do so. If universities are limited in their ability to expand the diversity and size of their student base, then ETPs are not as important. However, if universities have more autonomy in this direction, then ETPs are a means to the end of attracting a wider base of students and growing the size of the university.

Staffing autonomy involves freedom over recruitment, salaries, dismissals, and promotions for senior academic and administrative staff. Staffing autonomy was found to be a predictor of EMI (*p* = 0.005). Our model predicts that an increase of one point of staffing autonomy above the mean increases the likelihood of public universities offering EMI by 1.4%, and a ten-point increase raises this likelihood to 13.2%. Autonomy over staffing decisions is crucial for EMI because offering EMI requires the freedom to attract talent capable of teaching in English. This necessitates autonomy in recruitment procedures and the ability to provide internationally competitive salaries.

Organisational autonomy concerns the leadership model of the university: the freedom to choose who steers the ship and which direction to take it. Organisational autonomy was found to be a significant predictor of EMI in public universities (*p* = 0.007), increasing organisational autonomy by one point above the mean predicts an increased likelihood of offering EMI of 3.5%, which raises to 48.2% for a ten-point increase. This dimension may be particularly important as it concerns the elite participants who decide on whether to adopt EMI. As noted in the introduction, the Polytechnic University of Milan decided to teach all postgraduate degree programmes in English, against the wishes of the lecturers, who brought a legal case against the university (Murphy & Zuaro, 2021). In this case, the implementation of EMI was imposed by the leadership of the university. Our data supports the view that a key aspect of autonomy required to offer EMI is freedom over the leadership model of the university, which would grant them the ability to adopt EMI as a strategic choice.

Financial autonomy did not predict EMI in public universities. The directionality of the effect, the predicted probabilities in Fig. 4, and the predicted count in Fig. 5 all suggested that increasing financial autonomy may predict more EMI-practising universities and more ETPs, although we cannot rule out the null hypothesis that there is no relationship. Notably, being financially autonomous does not imply being well funded, nor does autonomy over tuition fees imply a greater number of international students. Whilst there are differences between the financial structures of public and private HEIs in terms of funding sources and financial freedom, we do not find that financial autonomy is relevant to the provision of EMI. In other words, assuming that private HEIs typically have greater autonomy over their finances than public HEIs, our data suggests it is not the freedom over finances that makes the difference in terms of EMI. However, there are likely other financial and economic factors which affect EMI not captured by financial autonomy, including market forces such as student demand. This may be a case of missing variable bias whereby financial autonomy could be relevant if other financial drivers are considered.

It is also worth discussing the fact that our models were better at predicting EMI versus non-EMI than predicting the number of ETPs in EMI-offering institutions. We highlight two reasons why this might be the case. First, a face-value interpretation: measures of autonomy are more relevant to whether institutions are able to offer EMI or not, but less relevant to modelling more/less EMI within HEIs. Second, a data limitation: the measure of ETPs is limited as programmes vary in size and HEIs may find that they are able to offer one or two ETPs and then increase student numbers if they are faced with increased demand. Increasing class size might be preferable to seeking approval for new programmes, hiring new staff, and so on. Therefore, an institution may be able to offer more EMI without increasing programme numbers. Using a measure of ‘percentage/number of students studying in EMI’ as a dependent may be better suited to investigating the growth of EMI within institutions, rather than ETPs, as in the current study. This draws attention to one of the key challenges of EMI research: the (un)availability of data on EMI practices in higher education.

A further area of interest is English proficiency. EPI Score was found to be negative and significant in our zero-inflation models for all four dimensions and was close to significance for overall autonomy. In other words, EPI score typically predicts the offering of EMI. This partially supports the position that EMI may be associated with countries with higher English ability. However, there is the possibility of missing variable bias, where the effect of EPI on EMI is moderated by other factors such as multilingualism, motivation, and the demand for English skills.

## Conclusion

### Summary

This paper investigated the relationship between EMI in European higher education and institutional autonomy, a key component of steering at a distance governance reforms. The University Autonomy Scorecards operationalised institutional autonomy in the study, which conceptualise autonomy in similar terms to how autonomy is framed within the context of steering at a distance reforms. In our terms, we understand this as a form of ‘regulated’ autonomy. Multilevel regression was used to test the relationship between university autonomy and EMI, drawing on a comprehensive database of 1815 HEIs nested within 26 education systems.

We found that overall, academic, staffing, and organisational autonomy predicted the probability of offering EMI in public HEIs, whereas financial autonomy did not. In cases of significance, the odds ratio was greatest concerning overall autonomy. Interestingly, academic, staffing, and organisational autonomy are the dimensions which involve autonomy over participants, broadly categorised as students (academic autonomy), academic and administrative staff (staffing autonomy), and senior management (organisational autonomy). Our findings are the first to quantify the link between governance and language of instruction, an area which we identify as a blind-spot in higher education governance.

### Limitations

The study offers a broad-brush account of the relationship between autonomy and EMI. However, we acknowledge that there is ambiguity over the exact nature of this relationship, particularly concerning the mechanism between autonomy and EMI. Autonomy may be a moderator/mediator between globalisation pressures and EMI, or perhaps autonomy functions as a proxy for other more crucial aspects of neoliberal reform. Moreover, our study was based on the 2017 edition of the autonomy scorecards and therefore provides a snapshot of Europe at that time and may be less applicable to current autonomy conditions in Europe or indeed higher education contexts outside of Europe. Further research in the form of detailed case studies could be conducted to trace how autonomy may or may not lead to EMI and what conditions promote or hamper it (see, Hultgren et al., 2023; Thomas et al., 2024). It would be interesting to see whether these processes are unique to the European context, or if they replicate worldwide.

This study drew on *internationally advertised* ETPs as a measure of EMI. Prior studies have measured EMI using institution-level survey data (Wächter & Maiworm, 2008) as well as internationally advertised ETPs (Sandström & Neghina, 2017; Wächter & Maiworm, 2014). Both measures are limited in the fact that they may not capture all programmes that run, either due to not advertising programmes or survey non-response. However, Study Portals is the largest online portal of ETPs, which connects universities to prospective students worldwide, which would only miss programmes which HEIs are not actively advertising. Therefore, whilst Study Portals provides substantial up-to-date data on ETPs, we may be missing programmes from institutions that are uninterested in international recruitment, preferring to recruit local students. As such, our measure is closer to ‘internationally oriented’ EMI. This draws attention to one of the main challenges of EMI research: the lack of good record keeping on language of instruction practices. We hope that by drawing attention to this issue, more data will be generated which captures language of instruction.

### Implications

The gap that this research identified was the theorised link between institutional autonomy, a key component of steering at a distance reforms, and EMI in European higher education. To date, the presence of EMI in higher education has been attributed to The Bologna Process, internationalisation, and the commodification of higher education. Our research provides evidence for a novel explanation: that EMI can be attributed, at least in part, to higher education autonomy. We are not claiming that institutional autonomy is the *only* explanation. In fact, the link between institutional autonomy and EMI is underpinned by other factors, such as massification, an increasingly mobile global population, and English as a *lingua franca*. Our findings provide a generalisable context for research conducted on higher education and EMI within Europe.

Underlying the practice of EMI are a range of ethical, legal, and political implications. The current research may provide an evidence-base to inform political and legal disputes over the implementation of EMI in contexts similar to the Polytechnic of Milan, where lecturers brought a legal case against the university, and in the Netherlands, which is currently considering legislation that restricts EMI in higher education. Our research suggests that the liberalisation of higher education may pave the way for EMI and therefore efforts to restrict EMI, by necessity, may have to restrict institutional autonomy. And conversely, efforts towards the liberalisation of higher education can be expected to coincide with EMI within the European context.

We hope that this research can highlight the importance of language of instruction for those within higher education governance, whereby EMI may be an unintended consequence of neoliberal reforms. The link between governance and linguistic outcomes is a critically under-researched area of study, partially due to a lack of available data on language of instruction. We would encourage those collecting data on higher education trends to include data on language of instruction practices, either at the programme level or at the level of student and staff participation. Moreover, researching the relationship between governance and linguistic outcomes requires interdisciplinary collaboration to break new ground; we hope that this article can pave the way for further research in this space.

## Footnotes

1 Wächter and Maiworm (2014, p.131) caution that a tenfold increase is likely an over-estimation as their earlier studies in 2001 and 2007 likely undercounted ETPs compared to 2014. Likewise, a 50-fold increase in English-taught bachelor’s programmes is likely an over-estimation, as the data in the Sandström and Neghina (2017) study were collected from the data company Study Portals, which was founded in 2009. It is unlikely that Study Portals managed to capture all ETPs in Europe within the first few years of their founding. A more modest estimate, based on a comparison with Wächter and Maiworm’s data, suggests approximately an eightfold increase in English-taught bachelors between 2009 and 2017.

2 This bill is yet to be commented on in the academic literature, but is reported here: https://www.theguardian.com/education/2023/jun/20/netherlands-seeks-curbs-on-english-language-university-courses accessed January 2024.

3 We compared Poisson and negative binomial models and their zero-inflated and hurdle counterparts. We also included variants in terms of negative binomial family, nbinom1 and nbinom2 from the glmmTMB package (Brooks et al., 2017). We found that the zero-inflated negative binomial model (nbinom2) fit the data best in terms of Akaike information criterion (AIC) and satisfied model assumptions concerning zero-inflation, dispersion, collinearity, and convergence.

4 As the interaction concerns a continuous variable and a categorical variable, we sum the coefficients for the continuous variable and the interaction and exponent the result. This differs from the odds ratio for the interaction term alone.
